# Spatial Positioning of RET and H4 Following Radiation Exposure Leads to Tumor Development

**DOI:** 10.1100/tsw.2001.31

**Published:** 2001-05-01

**Authors:** Yuri E. Nikiforov

**Affiliations:** Department of Pathology, University of Cincinnatti, OH 45267-0529, USA

**Keywords:** radiation, thyroid cancer, chromosomal rearrangements, nuclear architecture

## Abstract

Exposure to ionizing radiation is a well-known risk factor for a number of human cancers, including leukemia, thyroid cancer, soft tissue sarcomas, and many others. Although it has been known for a long time that radiation exposure to the cell results in extensive DNA damage, including double strand DNA breaks, the exact mechanisms of radiation-induced carcinogenesis remain unknown. Recently, a large increase in incidence of thyroid cancer was observed in children exposed to radiation after the Chernobyl nuclear accident [1]. A high prevalence of chromosomal rearrangements involving the RET gene was found among these radiation-induced thyroid tumors [2,3]. As a result of such rearrangement, a portion of the RET gene is fused with another gene, typically with the H4 or ELE1. However, since the DNA targets of ionizing radiation are randomly distributed throughout the cell nucleus, the reason for predilection for the RET rearrangements in thyroid cells was unclear.

A recent study published in *Science* by Nikiforova et al. shows that the answer to this question lies in the understanding of how different chromosomal loci are positioned in the nuclear volume with respect to each other [4]. Thus, it appears that the preferential occurrence of the *RET* gene rearrangements in thyroid cells may be due in large part to the structural organization of the nucleus of normal human thyroid cells resulting in geometric proximity of the genes that participate in the rearrangement. Such proximity may make two DNA molecules vulnerable to simultaneous damage by radiation, possibly by a single radiation track, and the mis-joining of two closely spaced free DNA ends will result in the rearrangement.

It has been a long-standing controversy in the field of radiation biology about how close target chromosomal loci have to be for radiation-induced rearrangement to occur [5]. Two main hypotheses were that these regions come into close contact after radiation damage occurred (the “breakage first” hypothesis), or that exchanges occur only where close contact already exists (the “contact first” hypothesis) [6]. The “breakage first” hypothesis has always been the dominant theory since it was more consistent with results obtained in experimental systems.

Nikiforova and her colleagues used two-color fluorescence *in situ* hybridization and three-dimensional microscopy to analyze the spatial positioning of the *RET* and *H4* genes, the two genes that participate in radiation-induced rearrangement, in the nuclei of normal human thyroid cells [4]. They demonstrated that in 35% of interphase nuclei *RET* and *H4* were juxtaposed to each other. They also found that such proximity is cell-type specific since it also exists in lymphocytes, but is absent in mammary epithelial cells. These data strongly support the “contact first” hypothesis and suggest that spatial contiguity provide a structural basis for tissue-specific generation of chromosomal rearrangements and subsequent tumor development after radiation exposure.

It is likely that geometric proximity is a necessary prerequisite for different types of chromosomal rearrangements associated with radiation exposure, such as *ABL-BCR*

Contact info: Phone: 513–558–5798; Fax: 513–558–2289
E-mail: Yuri.Nikiforov@uc.edu
© 2001 with author.

fusion in chronic myeloid leukemia and acute lymphoblastic leukemia. Further understanding of this phenomenon may provide a basis for development of new therapeutic strategies aimed to diminish the risk or prevent the development of tumors in people subjected to accidental or medical therapeutic irradiation.

## References

1. Balter, M. (1995) Chernobyl's thyroid cancer toll. *Science*, **270**, 1758–1759.
2. Fugazzola, L., Pilotti, S., Pinchera, A., Vorontsova, T.V., Mondellini, P., Bongarzone, I., Greco, A., Stakhova, L., Butti, M.G., Demidchik, E.P., Pacini, F., and Pierotti, M.A. (1995) Oncogenic rearrangements of the RET proto-oncogene in papillary thyroid carcinomas from children exposed to the Chernobyl nuclear accident. *Cancer Res.* **55,** 5617–5620.
3. Nikiforov, Y.E., Rowland, J.M., Bove, K.E., Monforte-Munoz, H., and Fagin, J.A. (1997) Distinct pattern of ret oncogene rearrangements in morphologic variants of radiation-induced and sporadic thyroid papillary carcinomas in children. *Cancer Res.* **57,** 1690–1694.
4. Nikiforova, M.N., Stringer, J.R., Blough, R., Medvedovic, M., Fagin, J.A., and Nikiforov, Y.E. (2000) Proximity of chromosomal loci that participate in radiation-induced rearrangements in human cells. *Science* **290**,138–141.
5. Savage, J.R. (1996) Insight into sites. *Mutat. Res.* **366,** 81–95.
6. Savage, J.R.K. (2000) Cancer: proximity matters. *Science* **290**, 62–63.

| | | |
|---|---|---|
| Received: | March | 5, 2001 |
| Accepted: | March | 26, 2001 |
| Published: | May | 1, 2001 |
